# Identification and Fine-Mapping of Quantitative Trait Loci Controlling Plant Height in Central European Winter Triticale (×*Triticosecale* Wittmack)

**DOI:** 10.3390/plants10081592

**Published:** 2021-08-02

**Authors:** Johannes Trini, Hans Peter Maurer, Jan Eric Neuweiler, Tobias Würschum

**Affiliations:** 1 State Plant Breeding Institute, University of Hohenheim, 70599 Stuttgart, Germany; johannes.trini@uni-hohenheim.de (J.T.); jan.neuweiler@uni-hohenheim.de (J.E.N.); 2 Institute of Plant Breeding, Seed Science and Population Genetics, University of Hohenheim, 70599 Stuttgart, Germany; tobias.wuerschum@uni-hohenheim.de

**Keywords:** triticale, plant height, genome-wide association mapping, fine-mapping, blast, *Ddw1*, *Rht12*

## Abstract

The quantitatively inherited trait plant height is routinely evaluated in triticale breeding programs as it substantially influences lodging and disease susceptibility, is a main contributor to biomass yield, and is required to improve hybrid seed production by fine-tuning plant height in the female and male parental pools in hybrid breeding programs. In this study, we evaluated a panel of 846 diverse Central European triticale genotypes to dissect the genetic architecture underlying plant height by genome-wide association mapping. This revealed three medium- to large-effect QTL on chromosomes 5A, 4B, and 5R. Genetic and physical fine-mapping of the putative QTL revealed that the QTL on chromosome 5R most likely corresponds to *Ddw1* and that the QTL on chromosome 5A is likely to be *Rht12*. Furthermore, we observed a temporal trend in registered cultivars with a decreasing plant height during the past decades, accompanied by an increasing use of the height-reducing alleles at the identified QTL. In summary, our results shed new light on the genetic control of plant height in triticale and open new avenues for future improvement by breeding.

## 1. Introduction

Hexaploid triticale (×*Triticosecale* Wittmack) is a man-made cereal that combines the genomes of tetraploid durum wheat (*Triticum durum* L.) and diploid rye (*Secale cereal* L.) [1]. The first triticale cultivars were released in the 1970s [1] and since then the triticale acreage has grown to approximately four million hectares worldwide as of 2019 [2]. Today’s grain use is mainly restricted to animal fodder and bioethanol production [1,3,4]. Its biomass is used as fresh fodder [4] and more recently for biogas production due to a promotional policy in the European Union [5,6,7,8].

Knowledge about the genetic control of plant height is of interest in small-grain cereal breeding programs. Tall plants usually are more susceptible to lodging resulting in substantial grain yield losses, reduced quality, and high drying costs during the harvesting process [9,10,11]. They also have a less favorable harvest index and the tendency consequently was to breed for shorter types. However, taller genotypes are also associated with a reduced Fusarium head blight susceptibility [12,13,14] and generally show an increased biomass yield, for which plant height is an important component of biomass yield [15,16,17]. Biomass yield has gained increasing interest as a breeding goal in triticale breeding programs in recent years due to the high potential of triticale as a bioenergy crop [8,18]. In addition, hybrid breeding has shown potential in triticale, which requires the control of the plant height of the male and female parental components, as the male lines should be taller compared to their female mating partners in order to increase the efficiency of hybrid seed production [19]. Thus, adjustment of plant height is required in triticale breeding programs, but the direction depends on the breeding goals.

Plant height in small-grain cereals is considered a quantitatively inherited trait controlled by many minor as well as by a few major quantitative trait loci (QTL) [17,20,21,22]. However, some height-reducing loci, such as the *Rht-1* homoeoloci, show adverse pleiotropic effects on other developmental processes, such as root elongation, early seedling vigor, or coleoptile length, which has been extensively studied in wheat [23,24,25,26]. In wheat and rye, the founding species of triticale, a large number of height-reducing loci are known [21,27,28,29,30]. Of the major QTL located on the A or B genome that are used in wheat breeding and are therefore potentially suitable to be used in triticale breeding, *Rht-B1* located on chromosome 4B has previously been reported in spring triticale [31]. QTL mapping in bi-parental triticale families has revealed several QTL on different chromosomes, but most of them with only small effects [14,17,32]. The only major QTL identified in triticale so far is *Ddw1*, located on chromosome 5 of the rye genome [14,17].

Consequently, the aims of this study were to investigate the phenotypic variation, the genetic architecture, and long-term genetic trends of plant height in Central European winter triticale. In particular, our objectives were to (i) perform genome-wide association mapping in a diversity panel of 846 Central European triticale genotypes, (ii) fine-map the genomic regions showing significant associations with plant height and determine their co-location with known height-reducing loci from wheat and rye, and (iii) evaluate long-term genetic trends and the allele frequencies of the detected QTL in registered cultivars.

## 2. Results

This study was based on 846 diverse triticale lines comprising 129 registered cultivars and 717 advanced breeding lines. All genotypes were evaluated in multi-location field trials for plant height and in addition for their developmental stage (BBCH scale) at a time point when the majority of the lines were heading, as this trait is often associated with plant height. We observed significant variation for both traits and high heritabilities of 0.81 for plant height and 0.80 for the developmental stage (Table 1). Plant height ranged from 87.3 cm to 126.6 cm and the BBCH stage ranged from 46.3 (opening of the flag leaf sheath) to 59.8 (completion of ear emergence). The correlation between the two traits was 0.24 (*p* < 0.001).

**Figure 1:**
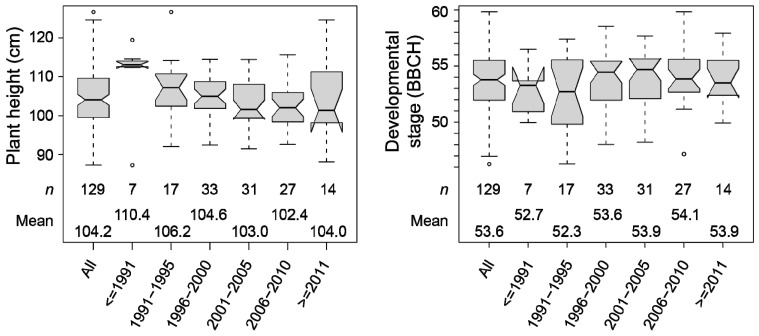
Development of plant height and developmental stage in the 129 registered cultivars dependent on their year of release.

We used the 129 registered cultivars to investigate long-term trends resulting from breeding in the period from 1982 when the first cultivars of this panel were released until today. Plant height declined in the period from 1982 to 2010 from on average 110.4 cm to 102.4 cm and showed an increasing proportion of taller genotypes from 2011 on (Figure 1). By contrast, the developmental stage did not change over time, except that there may be a slight tendency towards earlier heading in the more recent cultivars, i.e., a higher and thus more advanced BBCH stage.

**Figure 2:**
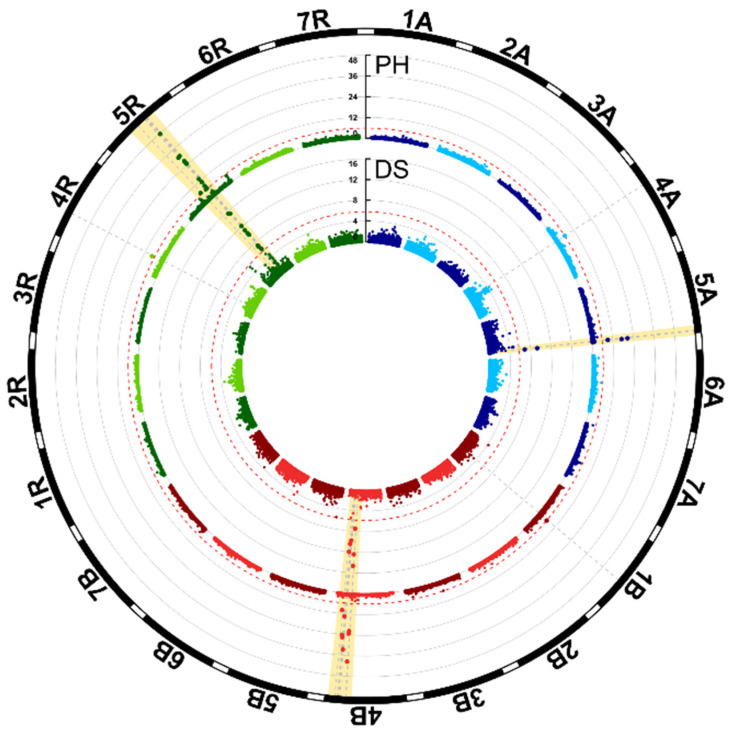
Circular Manhattan plot for plant height (PH) and developmental stage (DS). The red dashed lines indicate the Bonferroni-corrected significance threshold (*p* < 0.05) and the shaded area the QTL with pleiotropic effects on both traits.

We performed genome-wide association mapping using 31,823 markers to investigate the genetic architecture underlying the traits plant height and developmental stage (Figure 2). We identified 27 significantly associated markers for plant height and 17 for developmental stage (Supplementary Table S1). Jointly, these markers accounted for 42.16% of the total genotypic variance of plant height and 29.31% of that of developmental stage (Supplementary Table S1). The strongest association was found for the markers on chromosome 5R (Table 2). In a single fit, the QTL on 5A, 4B, and 5R explained more than 10% of the genotypic variance. In a joint fit, the QTL on chromosome 5R was fitted first and retained its high proportion of explained genotypic variance for plant height, whereas that of the 5A and 4B QTL was reduced to 0.40 and 2.34%, respectively. On chromosome 5R, three markers explained more than 1% of the genotypic variance even in a joint fit. This might indicate three separate QTL or that none of the markers alone is able to capture the full variance explained by a single QTL in this region. The fact that all three markers are located in close proximity, at 990.4, 997.0, and 1019.4 cm, suggests that they all identify the same QTL. Thus, the significantly associated markers might correspond to four QTL for plant height, located on chromosomes 5A, 4B, 4R, and 5R. The absolute values of the QTL allele substitution effects ranged between 1.03 and 5.30 cm (Table 2). Notably, the QTL effects varied substantially with the application rate of growth regulators and approximately doubled when only one instead of two rates were applied (Supplementary Figure S1). For the QTL on chromosome 5R, for example, the difference between the two allelic classes was 9.51 cm at the location Hohenheim when growth regulators were applied twice, which increased to 19.19 cm in the observation plots with only one application. For the developmental stage, three QTL were identified, the same QTL on chromosomes 5A, 4B, and 5R were also identified for plant height (Table 2). For these QTL, the allele that increased plant height also advanced the developmental stage, i.e., resulted in an earlier heading.

**Figure 3:**
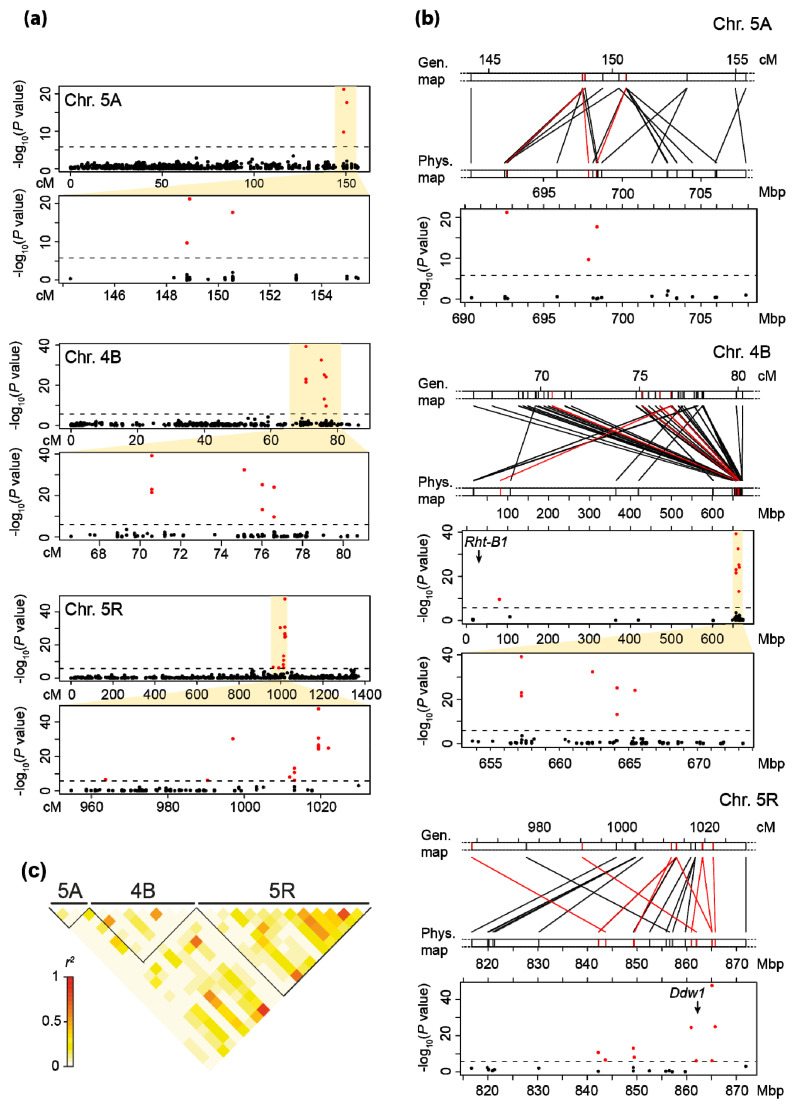
Fine-mapping of the identified QTL. (**a**) Genetic and (**b**) physical fine-mapping of the QTL on chromosomes 5A, 4B, and 5R. Red dots and lines indicate significantly associated markers after a Bonferroni multiple-test correction. (**c**) Linkage disequilibrium (*r*^2^) among the markers from the three QTL regions.

Next, we genetically and physically fine-mapped the plant height QTL on chromosomes 5A, 4B, and 5R (Figure 3a,b). The marker sequences were BLASTed against the respective reference genomes of wheat and rye to obtain their physical positions and to compare the QTL regions with those of known height-reducing loci. The significantly associated markers of the chromosome 5A QTL are located between 692 and 699 Mbp. For chromosome 4B, the significantly associated markers are located in the region between 657 and 666 Mbp, whereas *Rht-B1* is located at the front end of this chromosome. Physical map positions of all significant markers on chromosome 5R are located within the region from 842 to 875 Mbp. The dominant plant height locus *Ddw1* has recently been reported to be located at 862 Mbp [33]. Analysis of linkage disequilibrium (LD) among the significantly associated markers of these three QTL regions revealed a possible LD between some markers in the 4B and 5R regions (Figure 3c).

**Figure 4:**
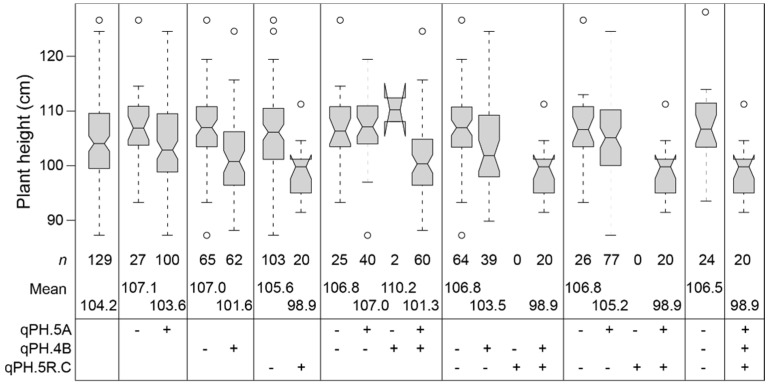
Effects of the QTL detected on chromosomes 5A, 4B, and 5R as well as their combinations on plant height, assessed in the 129 registered cultivars. ‘+‘ and ‘−’ indicate presence or absence of the height-reducing QTL alleles. If no presence/absence labeling is shown, the allelic state of the respective QTL was not considered.

**Figure 5:**
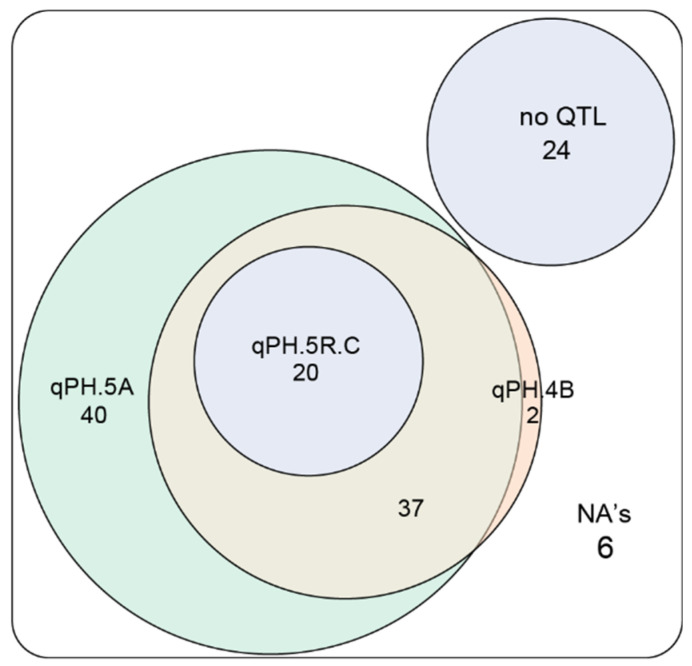
Number of cultivars with one or a combination of the plant height-reducing alleles at the detected QTL.

To better understand the effect of the detected QTL on plant height and their utilization in triticale breeding, we used the marker with the strongest association to represent the QTL on chromosomes 5A, 4B, and 5R (Figure 4, significantly associated markers and their allele status for the 129 registered cultivars can be found in the Supplementary Tables S2–S5). We found that in the 129 registered cultivars all three QTL occurred with both alleles and the difference between the two homozygous classes ranged between 3.5 and 6.7 cm. Notably, however, our analysis revealed that the height-reducing alleles often occur in combination (Figure 5). Twenty-four of the cultivars carry none of the height-reducing alleles at these three QTL, 97 carry the height-reducing allele at the 5A QTL, 59 at the 4B QTL, and 20 at the 5R QTL. The 20 cultivars carrying the height-reducing allele at the 5R QTL all also carry the height-reducing alleles at the 5A and 4B QTL. Likewise, the 59 cultivars carrying the height-reducing 4B QTL allele also carry the height-reducing allele at the 5A QTL, except for two. Besides the latter two cultivars, this leaves 40 that only carry the height-reducing allele at the 5A QTL, so at only one of the three QTL. The analysis of the advanced breeding lines and the complete panel showed similar results (Supplementary Figures S2–S4). The only difference compared to the subset of registered cultivars was the higher frequency in the whole panel of genotypes carrying all three QTL and the lower frequency of genotypes carrying only the QTL on chromosome 5A (Figure 5, Supplementary Figure S4).

**Figure 6:**
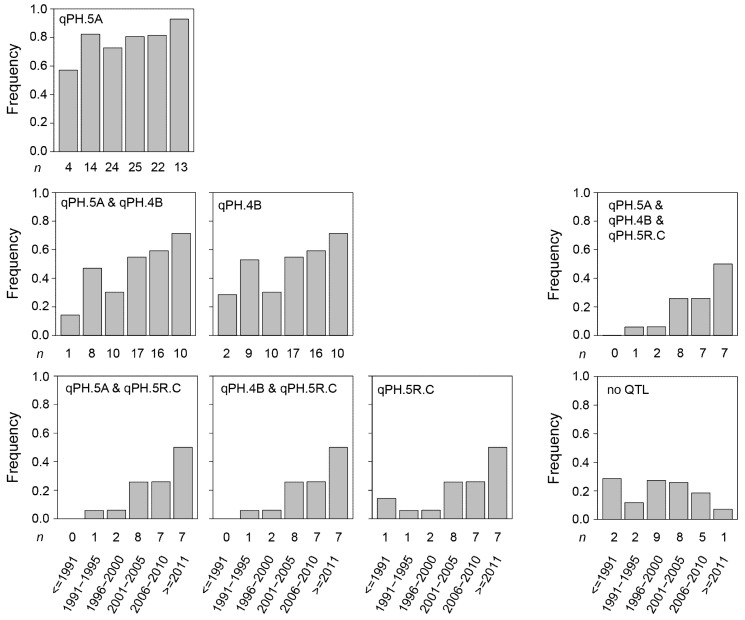
Temporal trends of the utilization of the identified QTL. Frequency of the height-reducing allele at the detected QTL qPH.5A, qPH.4B, and qPH.5R.C as well as their combinations in the registered cultivars dependent on their year of release.

Last, we investigated the development of the frequencies of the height-reducing alleles at these three QTL in registered cultivars over time. This revealed an increase for all three QTL, as well as an increasing frequency of their combinations (Figure 6). For the 5A QTL, the frequency of the height-reducing allele was already around 0.5 in the cultivars registered before 1991, which then increased to above 0.9 in the cultivars registered in the past decade. For the 4B QTL, the same frequency started at around 0.2 and then increased to around 0.6, and for the QTL on chromosome 5R the frequency of the height-reducing allele was low in the cultivars registered until the year 2000 and only then increased to around 0.5 in the cultivars registered since 2011.

## 3. Discussion

Plant height is of high importance in triticale breeding programs and in this study, we evaluated a diverse panel of 846 Central European triticale lines to investigate the genetic architecture underlying plant height, fine-map, and characterize identified QTL and draw conclusions for triticale breeding.

### 3.1. Genome-Wide Association Mapping and Characterization of QTL for Plant Height

Genome-wide association mapping revealed four putative QTL for plant height on chromosomes 5A, 4B, 4R, and 5R, of which the 5A, 4B, and 5R QTL can be considered as medium- to large-effect QTL. The QTL on chromosomes 5A, 4B, and 5R were also identified for developmental stage and thus have pleiotropic effects on both traits. The effects of these QTL on plant height may appear smaller than expected, but were shown to strongly depend on the application rate of growth regulators (Supplementary Figure S1). Notably, owing to the large variation present in the diversity panel, growth regulators were applied in these trials to prevent lodging and to mirror agronomic practice. Thus, the QTL effects can be expected to be larger in non-treated plants and reflect the substantial effect of the QTL even in combination with growth regulators.

In a single fit model with only one QTL, the 5A and 4B QTL explained 13.06 and 18.97% of the genotypic variance, respectively, which reduced to 0.40 and 2.34% in a joint fit of all QTL. Notably, the 5R QTL is fitted first in the joint fit model and thus retains its explained variance. The finding that the other two QTL do not, can have different reasons. It might be due to linkage disequilibrium among the QTL regions. However, at least for the 5A QTL, this LD with the 5R QTL was rather low and therefore LD among these QTL appears as a less likely explanation. A more likely reason is the joint occurrence of the height-reducing alleles of the three QTL in triticale lines observed in the registered cultivars but also in the entire panel (Figure 5). As the height-reducing allele of the 5R QTL occurs together with those of the 5A and 4B QTL, the genotypic classes are always confounded. Regarding the QTL on chromosomes 4B and 5R, the available genetic map positions as well as physical positions linked the significantly associated markers to regions on these two chromosomes. In an unrelated bi-parental population, however, we observed tight linkage among all these markers that segregated in that population (data not shown), indicating that they may actually identify only one QTL. Thus, further research using bi-parental families is required to disentangle the identities and effects of these QTL.

For the major QTL on chromosome 5R, three markers remained which in a joint fit still explained 1.79, 1.91, and 29.38% of the genotypic variance. While this could mean that there are three separate QTL, we concluded that it is more likely that they all identify the same QTL, as they are located in close proximity. Probably even the most strongly associated marker is not in perfect LD with the causal variant, such that also other markers can capture a small part of the variance explained by the QTL. Korzun et al. [34] mapped the dominant dwarfing gene *Ddw1* on the long arm of chromosome 5R. Braun et al. [33] recently identified a gibberellin 2-oxidase gene (*ScGA2ox12*) as a candidate for *Ddw1* in rye, which is located at 862 Mbp on chromosome 5R. As *Ddw1* has been reported in spring and winter triticale [14,17,31,35] and as the QTL region coincides with the position of *Ddw1*, we conclude that the 5R QTL corresponds to *Ddw1*. *Ddw1* is present in 20 of the 129 registered cultivars analyzed in this study and appears to have been increasingly used in recent years. However, previous work also illustrated adverse effects of *Ddw1*, for example on Fusarium head blight resistance [14]. The utilization of this height-reducing locus in triticale, therefore, requires further research on its advantages and limitations.

The QTL on chromosome 5A was located in the region between 692 and 699 Mbp (Figure 3b) and recently the dominant height-reducing *Rht12* locus has been reported to be located in the region between 700 and 710 Mbp of chromosome 5A in wheat [36]. It thus appears likely that the QTL on chromosome 5A corresponds to *Rht12*. To our knowledge, *Rht12* has not been reported in triticale germplasm so far, but its effect on plant height has been extensively investigated in wheat [23,37,38]. The height-reducing *Rht12* is reported to delay ear emergence in wheat [37,38] but this also depends on photoperiodic sensitivity loci [37]. We observed a pleiotropic effect of the QTL on chromosome 5A, with the allele that reduces plant height delaying development and resulting in later heading. Thus, further research is necessary to investigate the effects of the putative *Rht12* locus in triticale on other agronomic traits such as grain yield, ear emergence, disease resistance, early seedling vigor, or coleoptile length.

The height-reducing *Rht-B1* locus located on chromosome 4B has been investigated extensively in wheat [12,20,39,40,41], but much less so in triticale [31]. In this study, we identified a QTL on chromosome 4B with pleiotropic effects on plant height and developmental stage (Figure 2; Supplementary Table S1). However, *Rht-B1* is located on the short arm of chromosome 4B at approximately 30.8 Mbp, whereas the 4B QTL identified here is located at the opposite end of this chromosome (Figure 3b). Thus, *Rht-B1* is no candidate for this QTL, which is in line with previous findings in triticale, where *Rht-B1* did not contribute to the genetic control of plant height [32]. This QTL also showed a substantial plant height reduction and a high penetrance in the registered cultivars used in this study and is therefore of interest for future research.

### 3.2. Long-Term Trends of Plant Height in Triticale

The analysis of the development of plant height from the early 1980s—when the first cultivars of this panel were registered—until today, revealed a constant reduction in plant height (Figure 1) that is in line with previous findings [9]. In recent years, however, the demand for triticale genotypes with a greater biomass yield increased [15,42]. As plant height is one of the main contributors to biomass yield [15,16,17], taller genotypes were favored in breeding programs focusing on biomass yield.

In line with the phenotypic observation, we observed an increasing frequency of the height-reducing alleles at the three investigated QTL (Figure 6). The QTL on chromosome 5A, assumed to be *Rht12*, was constantly present at a medium to high frequency throughout the evaluated period. By contrast, especially the frequency of the QTL on chromosome 5R, likely *Ddw1,* increased only more recently since the turn of the millennium.

### 3.3. Conclusions

In this study, we identified three medium- to large-effect QTL and fine-mapping indicated that two of them correspond to *Rht12* and *Ddw1*. While *Ddw1* has been reported in triticale previously [14,17,31,35], *Rht12* has not yet been reported in triticale.

The widely used height-reducing locus *Rht-B1* has been one of the main contributors to improving grain yield and yield stability in wheat [20,39,43], but can negatively affect grain yield under drought conditions [43,44,45]. With the consequences of climate change [46], breeders will have to improve drought resistance also in triticale breeding material [47]. In wheat, the effect of height-reducing loci on seedling establishment and associated traits such as coleoptile length and early seedling vigor has been widely evaluated under dry conditions [25,26,48]. Genotypes carrying the dwarfing genes *Rht8* and *Rht12* have been reported to react less pronounced to heat and drought stress [23,37,49]. The effect of height-reducing loci on drought tolerance in triticale lacks behind what is known already in wheat and further research is required to evaluate the putative *Rht12* as well as other loci for their effects on drought tolerance and thus their potential for future breeding of climate-resilient triticale.

In conclusion, the diversity panel showed significant genotypic variation for plant height, which can be exploited to tailor plant height in breeding programs, either by phenotypic or marker-assisted selection. In addition, height-reducing loci from wheat, such as *Rht-B1* or *Rht24* [20,39], do not appear to be exploited in Central European triticale so far and could be introgressed from wheat.

## 4. Materials and Methods

### 4.1. Phenotypic Data

This study was based on a total of 846 triticale (×*Triticosecale* Wittmack) genotypes consisting of a diverse collection of officially registered Central European cultivars (*n* = 129) and lines in advanced breeding status (*n* = 717, for further details see Neuweiler et al. [50]). Phenotypic data of the diversity panel were taken from a large field trial (*n* = 1280), for which genotypes were grown in two separate sets comprising 800 (set A) and 500 (set B) individuals including 20 common checks. All genotypes were planted in yield plots (Y) with a plot size ranging from 5 to 10.5 m² and in addition, in double row observation plots (O) in the growing seasons 2014 and 2015. All field trials were designed as partially replicated α-lattice designs [51]. The yield plot trials had an average replication number of 1.3 and 1.2 for set A and B, respectively, observation plots were replicated twice. The field trials were grown in Eckartsweier (EWE, Y, 48°31′18′′ N, 7°52′18′′ E, 140 m above sea level, masl), Franconia (FRA, Y, 2014: 49°39′58′′ N, 9°47′30′′ E, 310 masl, 2015: 49°49′22′′ N, 10°6′19′′ E, 270 masl), Hohenheim (HOH, O, Y, 48°28′49′′ N, 9°11′16′′ E, 400 masl), Ihinger Hof (IHO, O, Y, 48°44′40′′ N, 8°55′25′′ E, 480 masl), Moosburg (MSB, Y, 48°26′36′′ N, 11°45′22′′ E, 420 masl), and Oberer Lindenhof (OLI, O, 48°28′49′′ N, 9°18′56′′ E, 700 masl) in 2014 and 2015 as follows: in 2014 only set A was evaluated as yield plots at the locations EWE, FRA, HOH, IHO, and MSB. In 2015, sets A and B were grown at HOH, IHO, and OLI as observation plots and in EWE, FRA, HOH, IHO, and MSB as yield plots. Genotypes grown in yield plots were treated with growth regulators twice and in observation plots once to prevent lodging. Plant height (cm) was assessed after flowering from the ground to the tip of the ears, excluding awns. In addition, the developmental stage was scored on a BBCH scale according to Zadoks et al. [52] when the ears of the majority of the genotypes were emerging.

### 4.2. Statistical Analysis

The linear mixed models used in this study followed the syntax as outlined by Piepho et al. [53], where dot operators specify crossed effects and fixed and random effects are separated by a colon, introducing fixed effects first. We used the following model to obtain best linear unbiased estimates (BLUEs) and least significant differences (LSD) as well as a full random model to determine variance components and heritability estimates:

*G:E + E·G + E·T + E·T·R·B + E·T·R·B* (1)

where *E*, *G*, *T*, *R*, and *B* denotes environments, genotypes, trial effects as a combination between genotype sets A and B and plot types (yield plots, double row observation plots), replications within each environment-trial combination, and incomplete blocks within replications of each environment-trial combination. Trial main effects were nested within environments. Variance components for the diversity panel used in this study were calculated using dummy variables in the above model. Variance components and heritabilities were estimated with the full random model using the restricted maximum likelihood method implemented in the software package ASReml-R 3.0 [54]. We assumed heterogeneous error variances at an environment-trial level. Variance components were tested for significance (*p* < 0.05, 0.01, 0.001) using a likelihood ratio test [55]. LSDs (*p* < 0.05) were calculated as an approximation using the twofold of the average standard error or a difference. Broad-sense heritability (*H*²) was estimated according to Cullis et al. [56] using the mean variance of a difference (${\bar{v}}_{BLUP}$) between two best linear unbiased predictors and the genotypic variance component ($\sigma_{G}^{2}$) as:

(2) $$H^{2}=1-\frac{{\bar{v}}_{BLUP}}{2{\;*\;\sigma }_{G}^{2}}$$

### 4.3. Molecular Data Analysis

Marker data were available for all of the 846 genotypes of the diversity panel and obtained by a genotyping-by-sequencing approach from Diversity Arrays Technology, Canberra, Australia (www.diversityarrays.com, accessed on 14 May 2021). Markers showing more than 20% missing values and a minor allele frequency of 5% or less were discarded. Unmapped significant markers were assigned to their most likely chromosomal position by their linkage disequilibrium with mapped markers. These measures yielded 31,823 markers in total that were used for the genome-wide association study. Positions for 10,192 markers on the A genome and 14,747 markers on the B genome were known [57]. Map positions for markers from the R genome were assigned as described in Neuweiler et al. [50] resulting in 6884 markers with a known map position. In total, 25,721 dominant silico-DArT and 6102 SNP markers were used. To differentiate between the silico-DArT and SNP markers, we assigned a “D” and an “S” prefix for silico-DArT and SNP markers, respectively. To determine the physical positions of markers in the QTL regions, the sequences of the markers were BLASTed against the reference genomes of wheat and rye (IWGSC RefSeq v1.0 and Secale cereale Lo7).

### 4.4. Genome-Wide Association Study

We performed genome-wide association mapping using a mixed linear model incorporating a kinship matrix [58] implemented in the GAPIT R package [59]. We used a Bonferroni-corrected significance threshold of *p* < 0.05. The genotypic data, filtered as stated above, were imputed using the k-nearest neighbor genotype imputation technique LD-kNNi implemented in LinkImpute [60]. Furthermore, we calculated the total proportion of the genotypic variance (*p_G_*) explained by all detected QTL as *p_G_ = R*^2^*_adj_/H*^2^, where *R*^2^*_adj_* was the adjusted *R*^2^ from the linear model, and *H*^2^ as the heritability of the trait [61]. To correct for collinearity, we ordered the QTL in the order of their strength of association and calculated their individual proportion of explained genotypic variance accordingly. The *p_G_* values for individual QTL were derived by estimating their sums of squares in the linear model. The allele substitution (α) effect was derived as α = *a*(1 + *k*(*p*_1_ − *p*_2_)) with *a* as genotypic value of the corresponding locus, *k* the degree of dominance, as well as *p*_1_ and *p*_2_ as the allele frequencies. The α-effect corresponds to one-half of the difference of the genotypic values of the corresponding genotypic classes of a QTL when inbred lines are considered. The α-effects were acquired by fitting a linear model for the marker of interest and the corresponding trait. Then the α-effect is represented by the determined regression coefficient [62].

## Figures and Tables

**Table 1:** Means and ranges of the best linear unbiased estimators as well as genotypic and genotype-by-environment interaction variance components of 846 diverse breeding lines and registered cultivars.

	Plant Height	Developmental Stage
Min	80.2	43.0
Mean	100.8	53.4
Max	126.6	59.8
LSD_0.05_	4.0	1.3
$\sigma_{G}^{2}$	77.98 ***	6.09 ***
$\sigma_{G\times E}^{2}$	7.38 ***	1.21 ***
$\sigma_{e}^{2}$ †	18.32	1.27
*H* ^2^	0.81	0.80

*** Significantly different from zero at the 0.001 probability level. † Mean residual error variance across environment-trial combinations.

**Table 2:** Results of the genome-wide association mapping for plant height and developmental stage.

QTL	Marker	Chr.	Pos. (cm)	*p*-Value	*p_G_* Joint ^a^	*p_G_* Single ^b^	*α*-Effect (Single Fit)	*p* ^c^
Plant height (42.16% *p_G_* total)								
qPH.5A	D10506872	5A	150.6	2.3 × 10^−18^	0.40	13.06	3.72	0.25
qPH.4B	D4371530 ^d^	4B	70.6	1.2 × 10^−23^	2.34	18.97	−4.02	0.36
qPH.4R	D10523748	4R	173.0	7.7 × 10^−8^	1.99	1.35	1.03	0.48
qPH.5R.A	D10519777	5R	990.4	7.3 × 10^−7^	1.79	4.56	2.26	0.23
qPH.5R.B	D4348428	5R	997.0	5.5 × 10^−31^	1.91	19.12	−3.94	0.41
qPH.5R.C	S4341499 ^d^	5R	1019.4	2.0 × 10^−48^	29.38	29.38	5.30	0.63
Developmental stage (29.31% *p_G_* total)								
qEC.5A	D10506872	5A	150.6	3.3 × 10^−10^	3.26	16.38	1.15	0.25
qEC.4B	D4372007	4B	70.6	2.7 × 10^−15^	0.03	12.83	−0.88	0.51
qEC.5R.B	D4348428	5R	997.0	2.4 × 10^−16^	16.74	16.74	−1.02	0.37
qEC.5R.C	S4341499 ^d^	5R	1019.4	4.1 × 10^−16^	5.35	19.43	1.19	0.63

^a^ *p_G_* values obtained by a joint fit of all significant markers for the respective trait in a linear model; markers were ordered according to their *p*-value (lowest first). ^b^ *p_G_* values obtained when each significant marker was fitted in a linear model for the respective trait. ^c^ Frequency of trait-increasing allele, i.e., development stages (for earlier genotypes), plant height (for taller genotypes). ^d^ Unmapped marker that was assigned to its most probable position based on its LD with mapped markers.

## Data Availability

The study did not report any data.

## Supplementary Materials

The following are available online at https://www.mdpi.com/article/10.3390/plants10081592/s1, Figure S1: Effects of the QTL qPH.5A, qPH.4B, and qPH.5R.C shown for observation and yield plots at Hohen-heim and Ihinger Hof. Observation plots were treated once, yield plots twice with growth regulators. The height-reducing QTL allele is shown in blue. Figure S2: Effects of the QTL detected on chromosomes 5A, 4B, and 5R as well as their combinations on plant height, assessed in the 846 registered cultivars and advanced breeding lines. ‘+’ and ‘−’ indicate presence or absence of the height-reducing QTL alleles. If no presence/absence labeling is shown, the allelic state of the respective QTL was not considered. Figure S3: Effects of the QTL detected on chromosomes 5A, 4B, and 5R as well as their combinations on plant height, assessed in the 717 advanced breeding lines. ‘+’ and ‘−’ indicate presence or absence of the height-reducing QTL alleles. If no presence/absence labeling is shown, the allelic state of the respective QTL was not considered. Figure S4: Number of genotypes carrying one or a combination of plant height reducing alleles at the detected QTL for the whole population including registered cultivars and advanced breeding lines (N = 846, left) as well as for the ad-vanced breeding lines alone (N = 717, right). Table S1: Significant markers detected for plant height and development stage by the genome wide association study.
