# Supplementary material for: Identification and Fine-Mapping of Quantitative Trait Loci Controlling Plant Height in Central European Winter Triticale (×Triticosecale Wittmack)
Source: Plants (Basel). 2021 Aug 2;10(8):1592. doi: 10.3390/plants10081592 (PMC8400435; doi:10.3390/plants10081592)
Supplement: Supplementary file 1 [file plants-10-01592-s001.zip › Supplementary_File_1_Review1.pdf]

plants

MDPI

Article

Identification and fine-mapping of quantitative trait loci controlling plant height in Central European winter triticale (×*Triticosecale* Wittmack)

Johannes Trini[1], Hans Peter Maurer[1*], Jan Eric Neuweiler[1], Tobias Würschum[2]

[1] State Plant Breeding Institute, University of Hohenheim, 70599 Stuttgart, Germany

[2] Institute of Plant Breeding, Seed Science and Population Genetics, University of Hohenheim, 70599 Stuttgart, Germany

* Corresponding author, E-mail: h-p.maurer@uni-hohenheim.de

Supplementary Material

Marker	Chr.	Pos. (cM)	p-value	p_G joint[a]	p_G single[b]	α-Effect (single fit)	p[c]
			Plant height (42.16% p_G total)				
D10521630	4A	83.9	3.4e-07	0.30	0.02	-0.26	0.06
S10514966	5A	148.8	2.0e-10	0.12	0.48	-0.78	0.78
D4354558	5A	148.9	6.8e-22	0.44	0.60	-0.69	0.45
D10506872	5A	150.6	2.3e-18	0.40	13.06	3.72	0.25
S4370961[d]	1B	102.8	1.0e-06	0.41	0.08	-0.31	0.33
D4371530[d]	4B	70.6	1.2e-23	2.34	18.97	-4.02	0.36
D4372007	4B	70.6	6.5e-40	0.00	19.40	-3.90	0.51
D3610345	4B	70.6	3.5e-22	0.38	0.01	0.16	0.93
D4545201	4B	75.1	4.1e-33	2.49	0.00	-0.02	0.37
D4554703	4B	76.0	6.9e-26	0.06	12.38	-3.15	0.43
D3609297	4B	76.0	8.1e-14	0.00	7.13	2.43	0.39
D10509945	4B	76.6	1.0 e-24	0.40	0.65	0.74	0.37
S4354727	4B	76.6	2.5e-10	0.05	0.26	0.53	0.39
D10523748	4R	173.0	7.7e-08	1.99	1.35	1.03	0.48
D10508815	5R	963.7	2.7e-07	0.23	0.85	-1.2	0.87
D10519777	5R	990.4	7.3e-07	1.79	4.56	2.26	0.23
D4348428	5R	997.0	5.5e-31	1.91	19.12	-3.94	0.41
S10520252	5R	1011.9	8.6e-09	0.06	4.59	1.99	0.49
S4370510	5R	1013.1	1.8e-11	0.33	10.00	3.15	0.41
D4341499[d]	5R	1013.1	6.9e-07	0.28	2.57	-1.80	0.19
S3045841	5R	1013.1	7.5e-14	0.12	0.15	0.61	0.1
S4341499[d]	5R	1019.4	2.0e-48	29.38	29.38	5.30	0.63
D3615774[d]	5R	1019.4	2.4e-31	0.02	16.68	-3.62	0.48
S3044752[d]	5R	1019.4	9.9e-27	0.01	19.39	-4.45	0.64
S3046128[d]	5R	1019.4	2.3e-27	0.19	20.32	-4.39	0.63
S4216997	5R	1019.4	2.6e-25	0.00	18.75	4.29	0.63
D10506889	5R	1022.0	1.1e-25	0.39	15.54	-3.50	0.46
			Developmental stage (29.31% p_G total)				
D4354558	5A	148.9	8.7e-08	0.79	0.48	-0.17	0.45
D10506872	5A	150.6	3.3e-10	3.26	16.38	1.15	0.25
D4371530[d]	4B	70.6	4.6e-08	0.14	8.27	-0.73	0.36
D4372007	4B	70.6	2.7e-15	0.03	12.83	-0.88	0.51
D3610345	4B	70.6	4.8e-13	0.09	0.26	-0.25	0.07

D4545201	4B	75.1	2.2e-10	0.00	1.43	0.30	0.93
D4554703	4B	76.0	4.6e-11	0.53	8.27	0.72	0.37
D10509945	4B	76.6	1.3e-12	0.01	0.47	0.17	0.39
D4348428	5R	997.0	2.4e-16	16.74	16.74	-1.02	0.37
S4370510	5R	1013.1	5.6e-08	0.08	8.85	0.82	0.41
S3045841	5R	1013.1	1.2e-07	0.02	0.52	0.32	0.41
S4341499[d]	5R	1019.4	4.1e-16	5.35	19.43	1.19	0.63
D3615774[d]	5R	1019.4	8.6e-12	1.03	16.79	-1.00	0.48
S4216997	5R	1019.4	4.4e-11	1.35	17.95	1.16	0.10
S3044752[d]	5R	1019.4	5.7 e-07	0.95	9.48	-0.86	0.46
S3046128[d]	5R	1019.4	1.2e-10	0.43	11.34	-0.91	0.64
D10506889	5R	1022.0	7.4e-13	0.02	12.91	-0.88	0.63

[a] p_G values obtained by a joint fit of all significant markers for the respective trait in a linear model; markers were ordered according to their *p*-value (lowest first).

[b] p_G values obtained when each significant marker was fitted in a linear model for the respective trait.

[c] Frequency of trait-increasing allele, *i.e.* development stages (for earlier genotypes), plant height (for taller genotypes).

[d] Unmapped marker that was assigned to its most probable position based on its LD with mapped markers.

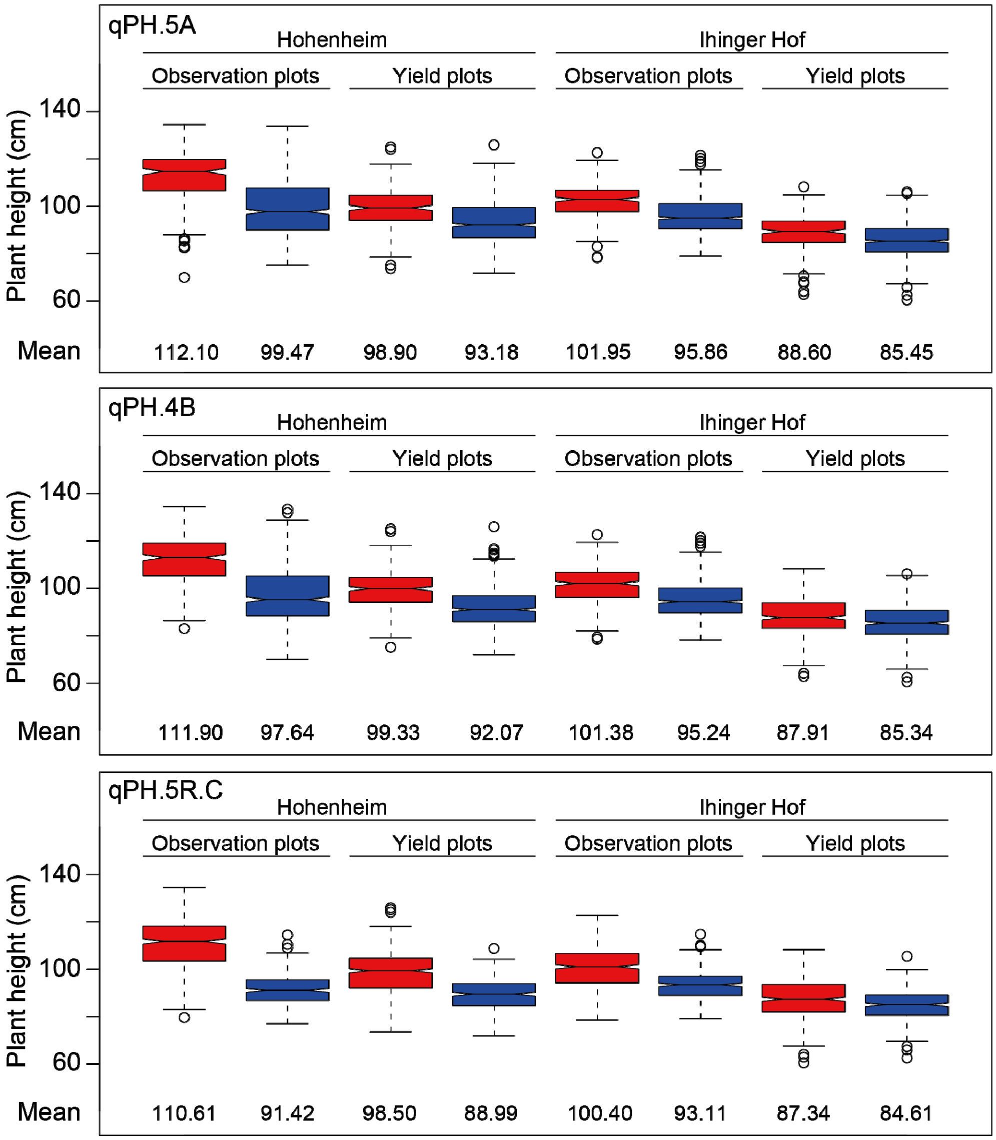

Supplementary Figure S1: Effects of the QTL qPH.5A, qPH.4B, and qPH.5R.C shown for observation and yield plots at Hohenheim and Ihinger Hof. Observation plots were treated once, yield plots twice with growth regulators. The height-reducing QTL allele is shown in blue.

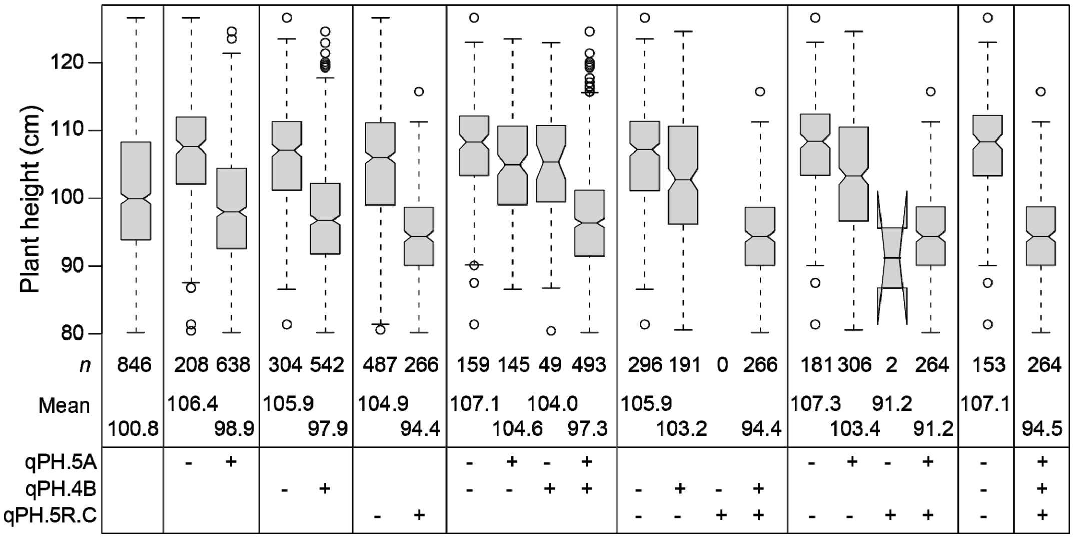

Supplementary Figure S2: Effects of the QTL detected on chromosomes 5A, 4B, and 5R as well as their combinations on plant height, assessed in the 846 registered cultivars and advanced breeding lines. '+' and '-' indicate presence or absence of the height-reducing QTL alleles. If no presence / absence labeling is shown, the allelic state of the respective QTL was not considered.

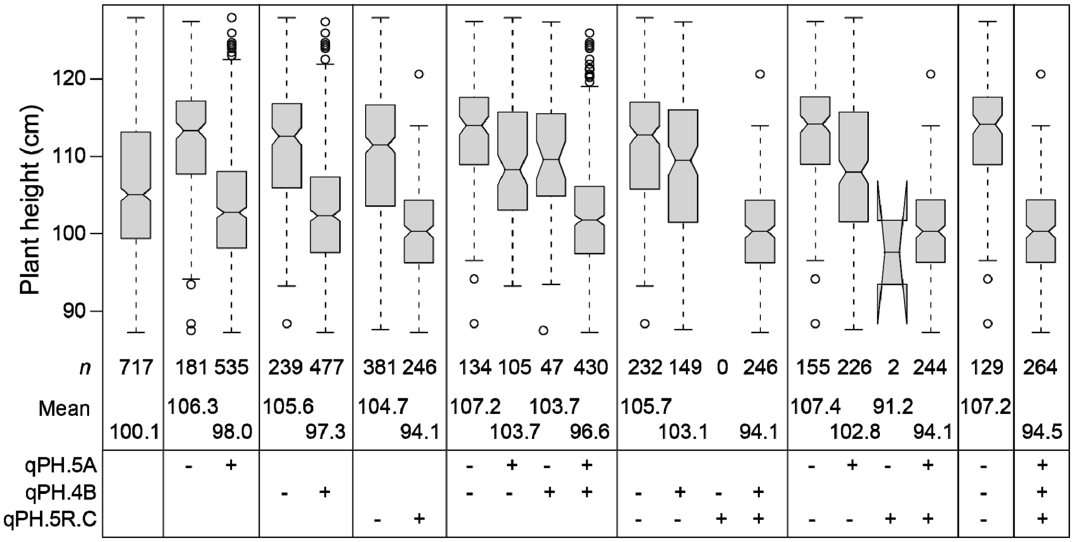

Supplementary Figure S3: Effects of the QTL detected on chromosomes 5A, 4B, and 5R as well as their combinations on plant height, assessed in the 717 advanced breeding lines. '+' and '-' indicate presence or absence of the height-reducing QTL alleles. If no presence / absence labeling is shown, the allelic state of the respective QTL was not considered.

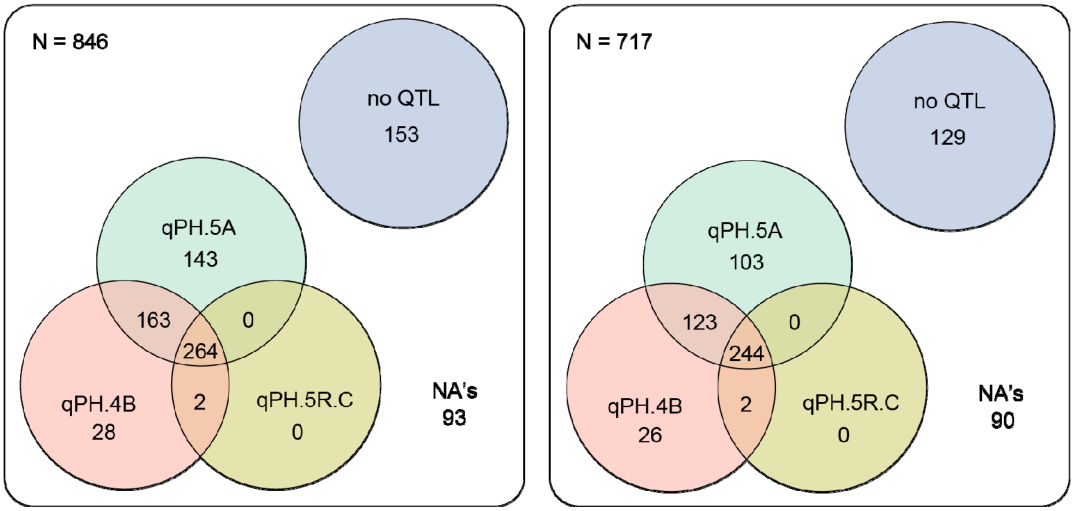

Supplementary Figure S4: Number of genotypes carrying one or a combination of plant height reducing alleles at the detected QTL for the whole population including registered cultivars and advanced breeding lines (N = 846, left) as well as for the advanced breeding lines alone (N = 717, right).
